# 3D MHD nonlinear radiative flow of CuO-MgO/methanol hybrid nanofluid beyond an irregular dimension surface with slip effect

**DOI:** 10.1038/s41598-020-66102-w

**Published:** 2020-06-08

**Authors:** Iskander Tlili, Hossam A. Nabwey, S. P. Samrat, N. Sandeep

**Affiliations:** 1 Department for Management of Science and Technology Development, Ton Duc Thang University, Ho Chi Minh City, Vietnam; 2 Faculty of Applied Sciences, Ton Duc Thang University, Ho Chi Minh City, Vietnam; 3 Department of Mathematics, College of Science and Humanities in Al-Kharj, Prince Sattam bin Abdulaziz University, Al-Kharj, 11942 Saudi Arabia; 4 Department of Basic Engineering Science, Faculty of Engineering, Menoufia University, Shebin El-Kom, 32511 Egypt; 5 Department of Mathematics, Central University of Karnataka, Kalaburagi, 585367 India

**Keywords:** Computational chemistry, Applied mathematics

## Abstract

The 3D MHD nonlinear radiative hybrid nanofluid flow across an irregular dimension sheet with slip effect is studied numerically. The hybrid nanofluid consists of copper oxide (CuO) and magnesium oxide (MgO) nanoparticles embedded in methanol or methyl alcohol (MA). The governing PDEs’ are altered as ODEs’ using similarities and numerical solutions are attained using shooting scheme. The role of corporal factors on the transport phenomenon is analyzed and reflected by plots and numerical interpretations. Simultaneous solutions presented for CuO-MA nanofluid and CuO-MgO/MA hybrid nanofluid. Results ascertain that the temperature and flow boundary layer thicknesses are not unique for the hybrid nanofluid and nanofluid. The heat transfer enactment of CuO-MA nanofluid is high when equated to CuO-MgO/MA hybrid nanofluid. This concludes that the CuO-MgO combination works as a good insulator.

## Introduction

The novelty of discoveries in heat transport to stimulate the efficiency of hybrid nanofluid with several assortments of conductive nanoparticles has wide applications in engineering and technology. There is a tiny evolution for hybrid nanofluid studies in recent days. In 1995, the nanofluids were developed and got changes in the efficiency of heat transfer drastically^1^. Generally, the nanofluid is a synthesis of mono-nanoparticle in base liquid to promote superior thermal liquids. The crises of cooling demand can be cracked by evolving nanofluid were reported by Kumar *et al*.^2^. The advancement in nanofluid is a hybrid-nanofluid to enrich more heat transfer. The fusion of two or more than two nanoparticles in a base liquid will get a hybrid nanofluid^3^. Several scholars have pointed out that the assets of hybrid nanofluid are greater as associated with nanofluid^4–6^. These fluids are mostly used in renewable energy, emollients, air conditioning, microelectronics, etc^7^.. Yarmand *et al*.^8^ deliberate the fusion of platinum hybrid nanofluids and clarified its physical properties and stability. The pressure drop of these solutions is smaller than that of nanofluid or any base liquid, so that it has auspicious applications that were suggested by Huang *et al*.^9^.

Later on, Labib *et al*.^10^ considered the CNT-Al2O3 hybrid nanofluid and originated the mix of Al2O3-CNT nanoparticle in base-liquid to improve the convection as equated to Al2O3-nanoliquid. Waini *et al*.^11^ discussed the unsteady flow above a widening area and established the stability of the heat transfer of hybrid nanofluid, which is depending on the miscellany of the nanoparticle. Ma *et al*.^12^ stated the bearings of MHD on hybrid nanofluid in a channel with powerful heater and cooler walls. They invented that heat transmission is more active in the heater wall as related to the cooler wall. Sheikholeslami *et al*.^13^ considered the heat transmission of magnetizable hybrid nanofluid in a circular cavity and theoretically showed that the strength of magnetic fields located in circular heaters and MWCNT-Fe3O4 with base liquid would maximize the heat transfer rate. The hybrid nanofluid past a spongy medium was described by Mehryan *et al*.^14^. They found that the sponginess ratio will augment the convective flow inside the cavity. Acharya *et al*.^15^ exhibited the effects of thermodiffusion on hybrid-nanofluid past a spinning disk and initiated that the radiation factor was escalations the heat transfer rate. Using the lattice Boltzmann method, Reza and Shahriari^16^ examine the assets of the magnetic field in existence of Rayleigh number and pointed out that Hartmann number digresses in the absences of Rayleigh number.

The heat transport in the flow over an elongated sheet has widely used in manufacturing process, polymer extrusion, paper fabrication, hot rolling process, aeronautical, civil and marine engineering, etc. Moreover, the consideration of variable thickness is to develop an essential and consistent design. Fang *et al*.^17^ explored the importance of variable thickness geometry. Khader and Megahed^18^ examined the boundary layer movement over a nonlinear elongating plate and found that the heat rate enriches due to movable thickness. Khan *et al*.^19^ studied Carreau nanofluid over an elongating sheet, solved numerically under some influences of thermal radiation and movable thickness property. The behavior of the movement of the heat of dusty nanofluids over an extending superficial was considered by Sandeep *et al*.^20^. Hayat *et al*.^21^ studied the boundary layer flow with homogeneous-heterogeneous reaction and found that the property of movable thickness indicates acceleration in flow rate. The 3-D slip flow above an elongating sheet with cross-diffusion was adopted by^22^ and elucidated that the uneven thickness stretching sheet enhances the energy. The authors^23,24^ considered the MHD flow across a slender surface and moving the needle and examined the transport phenomena.

Furthermore, MHD effects are used in metal casting, metallurgy, the invention of medicine, extraction of geothermal energy, polymer industry, melting reactors, fusion reactor, etc. Sulochana *et al*.^25^ measured the flow over a revolving cone with magnetic and Soret effects. Usman *et al*.^26^ examines heat flow assets of Al2O3-Cu-hybrid nanofluid with magnetic and radiation effects. Nayak *et al*.^27^ exposed that the existence of MHD slowdowns the fluid motion and escalations of thermal profile. Khan *et al*.^28^ explained the rotating flow of hybrid nanofluid under magnetic effects. Reddy *et al*.^29^ analyzed the MHD nanofluid flow through the spongy passage by the perturbation technique. Samrat *et al*.^30^ scrutinized an unsteady flow of Casson fluid under the magnetic and radiation effects over an elongating surface. Conduct of hybrid nanofluid in a wavering upright channel with hall current was scrutinized by Iqbal *et al*.^31^. Recently, the researchers^32–34^, numerically investigated the convective heat transfer in magnetohydrodynamic flows past a stretched surface with non-uniform thickness in the presence of various physical effects. The impact of Lorentz force and slip effect on free convection flow past a annulus was numerically investigated by the researchers^35–37^. Further, Jagadeesha *et al*.^38^ studied the effect of magnetic field on the flow through a porous enclosure. Later on, the researchers^39,40^ discussed the influence of buoyancy and magnetic field on electrically conducting fluid past a vertical annulus.

By keeping the above developments in view, we portrayed the convective heat transmission in hybrid nanofluid drift over the variable dimension elongating sheet. For this, we considered the magnetohydrodynamic nonlinear radiative CuO-MgO/MA hybrid nanofluid with velocity slip and temperature jump. Numerical solutions are attained using shooting scheme. The role of corporeal factors on the transport phenomenon is analyzed and reflected by plots and numerical interpretations. Simultaneous solutions are presented for CuO-MA nanofluid and CuO-MgO/MA hybrid nanofluid.

## Mathematical Formulation

**Figure 1:**
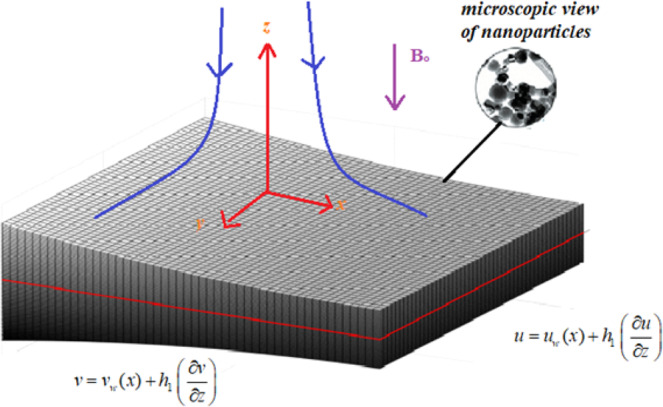
Schematic Model.

A steady, 3-D electrically conducting magnetohydrodynamic flow of CuO-MgO/MA hybrid nanofluid past an extended surface of non-uniform thickness is considered. The sheet of non-uniform thickness is considered as $z=A{\delta }^{(1-n)/2},\,\delta =x+y+c,n\ne 1$ we have chosen *A* is small. It is assumed that the surface is stretched along the *xy*-plane while fluid is placed along the $z$-axis. It is also presumed, the sheet temperature as ${T}_{w}={T}_{0}{\delta }^{\frac{1-n}{2}}+{T}_{\infty }$. The magnetic field of strength $B$ is applied in parallel with the $z-$ axis as revealed in Fig. 1.

With conventions made above, the governing equations can be expressed as:^11,17–19^

1 $${u}_{x}+{v}_{y}+{w}_{z}=0,$$

2 $${\rho }_{hnf}(u{u}_{x}+v{u}_{y}+w{u}_{z})={\mu }_{hnf}{u}_{zz}-{\sigma }_{hnf}{B}^{2}u,$$

3 $${\rho }_{hnf}(u{v}_{x}+v{v}_{y}+w{v}_{z})={\mu }_{hnf}{v}_{zz}-{\sigma }_{hnf}{B}^{2}v,$$

4 $${\rho }_{hnf}(u{T}_{x}+v{T}_{y}+w{T}_{z})={\mu }_{hnf}{T}_{zz}-{({q}_{r})}_{z},$$

with the frontier situations

5 $$\begin{array}{c}u-{u}_{w}={h}_{1}^{\ast }\left(\frac{\partial u}{\partial z}\right),v-{v}_{w}={h}_{1}^{\ast }\left(\frac{\partial v}{\partial z}\right),\\ T-{T}_{w}-{h}_{2}^{\ast }\left(\frac{\partial T}{\partial z}\right)=0,z=A{\delta }^{(1-n)/2},n\ne 1\\ and\,u(\infty )\to 0,v(\infty )\to 0,T(\infty )\to {T}_{\infty }\end{array}\}$$

where *u*,*v* are velocity components in *x* and *y* directions, ${u}_{w},{v}_{w}$ are velocities near surface, ${h}_{1}^{\ast },{h}_{2}^{\ast }$ are dimensional velocity slip and temperature jump parameters, ${T}_{W}$ is the wall temperature^32–34^

6 $$\begin{array}{c}{\zeta }_{1}=\frac{{k}_{B}T}{\sqrt{2}\pi {d}^{2}p},\delta =x+y+c,{h}_{1}^{\ast }=\left[\frac{2-{f}_{1}}{{f}_{1}}\right]{\zeta }_{1}{\delta }^{\frac{1-n}{2}},\\ {\zeta }_{2}=\left(\frac{2\gamma }{\gamma +1}\right)\frac{{\zeta }_{1}}{\Pr },{h}_{2}^{\ast }=\left[\frac{2-b}{b}\right]{\zeta }_{2}{\delta }^{\frac{1-n}{2}},B={B}_{0}{\delta }^{\frac{n-1}{2}},\end{array}\}$$

7 $${u}_{w}=a{\delta }^{\frac{n-1}{2}},{v}_{w}=a{\delta }^{n},{T}_{w}-{T}_{\infty }={T}_{0}{\delta }^{\frac{1-n}{2}},{\rm{for}}\,n\ne 1$$

By Rosseland approximation, the heat flux is given by

8 $${q}_{r}=-\frac{4{\sigma }^{\ast }}{3{k}^{\ast }}\left(\frac{\partial {T}^{4}}{\partial z}\right)$$

after expanding *T*^4^ through Taylor series about *T*_∞_ and neglecting higher-order terms and considering ${\theta }_{w}=\frac{{T}_{w}}{{T}_{\infty }}$, the resulting similarity variable of energy becomes

9 $$T={T}_{\infty }[1+({\theta }_{w}-1)\theta ]$$

The above novel form is involved for the parameterization of the nonlinear radiation term apparent in the energy equation. Where ${\sigma }^{\ast },{k}^{\ast }$ denotes the Stefan-Boltzmann constant and mean absorption coefficient.

The hybrid nanofluid parameters can be used as:^11^

10 $$\begin{array}{c}\frac{{k}_{hnf}}{{k}_{f}}=\frac{{k}_{2s}+2{k}_{f}-2{\phi }_{2}({k}_{f}-{k}_{2s})}{{k}_{2s}+2{k}_{f}+{\phi }_{2}({k}_{f}-{k}_{2s})}\times \frac{{k}_{1s}+2{k}_{f}-2{\phi }_{1}({k}_{f}-{k}_{1s})}{{k}_{1s}+2{k}_{f}+{\phi }_{1}({k}_{f}-{k}_{1s})},\\ \frac{{\rho }_{hnf}}{{\rho }_{f}}=(1-{\phi }_{2})\left[(1-{\phi }_{1})+\frac{{\phi }_{1}{\rho }_{1s}}{{\rho }_{f}}\right]+\frac{{\phi }_{2}{\rho }_{2s}}{{\rho }_{f}},\phi ={\phi }_{1}+{\phi }_{2},\\ \frac{{(\rho {c}_{p})}_{hnf}}{{(\rho {c}_{p})}_{f}}=(1-{\phi }_{2})\left[(1-{\phi }_{1})+\frac{{\phi }_{1}{(\rho {c}_{p})}_{1s}}{{(\rho {c}_{p})}_{f}}\right]+\frac{{\phi }_{2}{(\rho {c}_{p})}_{2s}}{{(\rho {c}_{p})}_{f}},\\ \frac{{\mu }_{hnf}}{{\mu }_{f}}=\frac{1}{{(1-{\phi }_{1})}^{2.5}{(1-{\phi }_{2})}^{2.5}},\frac{{\sigma }_{hnf}}{{\sigma }_{f}}=\left[1+\frac{3({\sigma }_{1s}{\phi }_{1s}-\phi {\sigma }_{f})+{\phi }_{2s}{\sigma }_{2s}}{{\sigma }_{1s}(1-{\phi }_{1s})+{\sigma }_{2s}(1-{\phi }_{2s})+(2+\phi ){\sigma }_{f}}\right],\end{array}\}$$

where *ϕ*_1_, *ϕ*_2_ denotes the CuO and MgO nanoparticle volume fractions. The following similarity transformations are used for non-dimensionalization^33^

11 $$\begin{array}{c}\eta =z{\left(\frac{(n+1)a}{2\upsilon }\right)}^{1/2}{\delta }^{(n-1)/2},\theta =(T-{T}_{\infty })/({T}_{w}-{T}_{\infty }),\\ u=a{\delta }^{n}f{\prime} (\eta ),v=a{\delta }^{n}g{\prime} (\eta )\\ w=-{\left(\frac{2a\nu }{n+1}\right)}^{0.5}{\delta }^{(n-1)0.5}\left[\frac{n+1}{2}(f+g)+\eta \left(\frac{n-1}{2}\right)(f{\prime} +g{\prime} )\right]\end{array}\}$$

by making use of Eqs. (6–9), the Eqs. (1–5) can be transformed as

12 $$\begin{array}{c}\frac{n+1}{2{(1-{\phi }_{1})}^{2.5}{(1-{\phi }_{2})}^{2.5}}f\prime\prime\prime -\left(1+\frac{3({\sigma }_{1s}{\phi }_{1s}-\phi {\sigma }_{f})+{\phi }_{2s}{\sigma }_{2s}}{{\sigma }_{1s}(1-{\phi }_{1s})+{\sigma }_{2s}(1-{\phi }_{2s})+(2+\phi ){\sigma }_{f}}\right)Mf{\prime} \\ -\left((1-{\phi }_{2})\left((1-{\phi }_{1})+\frac{{\phi }_{1}{\rho }_{1s}}{{\rho }_{f}}\right)+\frac{{\phi }_{2}{\rho }_{2s}}{{\rho }_{f}}\right)\left(n{(f{\prime} )}^{2}+nf{\prime} g{\prime} -\frac{n+1}{2}(f+g)f{\prime\prime} \right)=0,\end{array}\}$$

13 $$\begin{array}{c}\frac{n+1}{2{(1-{\phi }_{1})}^{2.5}{(1-{\phi }_{2})}^{2.5}}g\prime\prime\prime -\left(1+\frac{3({\sigma }_{1s}{\phi }_{1s}-\phi {\sigma }_{f})+{\phi }_{2s}{\sigma }_{2s}}{{\sigma }_{1s}(1-{\phi }_{1s})+{\sigma }_{2s}(1-{\phi }_{2s})+(2+\phi ){\sigma }_{f}}\right)Mg{\prime} \\ -\left((1-{\phi }_{2})\left((1-{\phi }_{1})+\frac{{\phi }_{1}{\rho }_{1s}}{{\rho }_{f}}\right)+\frac{{\phi }_{2}{\rho }_{2s}}{{\rho }_{f}}\right)\left(n{(g{\prime} )}^{2}+nf{\prime} g{\prime} -\frac{n+1}{2}(f+g)g{\prime} \right)=0,\end{array}\}$$

14 $$\begin{array}{c}\left(\begin{array}{c}\left(\frac{{k}_{2s}+2{k}_{f}-2{\phi }_{2}({k}_{f}-{k}_{2s})}{{k}_{2s}+2{k}_{f}+{\phi }_{2}({k}_{f}-{k}_{2s})}\right)\left(\frac{{k}_{1s}+2{k}_{f}-2{\phi }_{1}({k}_{f}-{k}_{1s})}{{k}_{1s}+2{k}_{f}+{\phi }_{1}({k}_{f}-{k}_{1s})}\right)\theta {\prime\prime} +R{(1+({\theta }_{w}-1)\theta )}^{3}\theta {\prime\prime} +\\ \,3R{(1+({\theta }_{w}-1)\theta )}^{2}({\theta }_{w}-1){\theta {\prime} }^{2}\end{array}\right)\\ \,-\frac{2\Pr }{n+1}\left((1-{\phi }_{2})\left[(1-{\phi }_{1})+\frac{{\phi }_{1}{(\rho {c}_{p})}_{1s}}{{(\rho {c}_{p})}_{f}}\right]+\frac{{\phi }_{2}{(\rho {c}_{p})}_{2s}}{{(\rho {c}_{p})}_{f}}\right)\left(\frac{1-n}{2}(f{\prime} +g{\prime} )\theta -\frac{n+1}{2}(f+g)\theta {\prime} \right)=0,\end{array}\}$$

the transmuted boundary restrictions are

15 $$\begin{array}{c}f(0)=\lambda \left(\frac{1-n}{n+1}\right)[1+{h}_{1}f{\prime\prime} (0)],f{\prime} (0)=[1+{h}_{1}f{\prime\prime} (0)],\\ g(0)=\lambda \left(\frac{1-n}{n+1}\right)[1+{h}_{1}g{\prime\prime} {(\eta )}_{\eta =0}],\theta (0)=[1+{h}_{2}\theta {\prime} (0)],\\ g{\prime} (0)=[1+{h}_{1}g{\prime\prime} (0)],f{\prime} {(\eta )}_{\eta \to \infty }=0,g{\prime} {(\eta )}_{\eta \to \infty }=0,\theta {(\eta )}_{\eta \to \infty }=0,\end{array}\}$$

where

16 $$M=\frac{{\sigma }_{f}{B}_{0}^{2}}{{\rho }_{f}a},\Pr =\frac{{\mu }_{f}{({c}_{p})}_{f}}{{k}_{f}}$$

are the magnetic field parameter and Prandtl number and $\lambda$ is wall thickness parameter.

For engineering curiosity the ${C}_{f}$ and $N{u}_{x}$ are defined as^22^

17 $${C}_{f}=2\frac{{\mu }_{hnf}}{{\mu }_{f}\sqrt{\mathrm{Re}}}{\left(\frac{n+1}{2}\right)}^{0.5}{f{\prime\prime} |}_{\eta =0},N{u}_{x}=-\sqrt{\mathrm{Re}}(1+R{\theta }_{w})\frac{{k}_{hnf}}{{k}_{f}}{\left(\frac{n+1}{2}\right)}^{0.5}{\theta {\prime} |}_{\eta =0}\}$$

where $$\mathrm{Re}=\frac{{u}_{w}\delta }{{\upsilon }_{f}},$$

### Discussion of the results

The system of Eqs. (12–14) are solved by shooting scheme under the limitations of Eq. (15). We discussed the eccentricities of velocity and temperature with impacts of non-dimensional parameters through graphs. The velocity power index *n*, thermal radiation *R*, temperature ratio ${\theta }_{w}$, wall thickness $\lambda$, velocity slip ${h}_{1}$, temperature jump ${h}_{2}$, and nanoparticle volume fraction $\phi (={\phi }_{1}+{\phi }_{2}),{\phi }_{1}$ parameters are used in this study. Also, the friction factor and Nusselt number are scrutinized with the above said parameters through the table. The physical parametric values are set to $M=1,n=0.7,{h}_{1}=0.4,{\theta }_{w}=0.3,R=1,{h}_{2}=0.4,\lambda =0.1,\Pr =7.38$ in order to attain the required results. These values are invariant unless the variations shown in the respective figures and tables. Table 1 predicts the thermo physical properties. Table 2 displays the authentication of the results. Tables 3 and 4 discuss the impact of pertinent parameters on flow and heat rate. The heat flow rate uplift and contradictory is seen in friction factor due to the variation in $\lambda$. Nusselt number is drastically reducing for rising values of thermal radiation. The same trend has been observed for boosting values of magnetic field and slip parameters.

**Table 1:** Thermo physical properties.

Property	Methanol	*CuO*	*MgO*
$\rho \,(Kg/{m}^{3})$	792	6320	3580
${C}_{p}\,(J/KgK)$	2545	531.8	960
$k\,(W/mK)$	0.2035	76.5	48.4
$\sigma (S/m)$	0.5 × 10^−6^	6.9 × 10^−2^	1.42 × 10^−3^

**Table 2:** Validation of the results for $f\text{'}\text{'}(0)$ (2D case-water with ϕ = 0) for various values of $\lambda$ and ${h}_{1}$ in the absence of radiation.

*h*_1_	*λ*	Ref. ^32^	Present Results
0	0.2	−0.924828	−0.92482831
0.2	0.25	−0.733395	−0.73339520
0.2	0.5	−0.759570	−0.75957013

**Table 3:** Effects of non-dimensional quantities on *f*″(0),–*θ*′(0) for Methanol+CuO+MgO.

*λ*	*R*	*θ*_*w*_	*n*	*ϕ*_1_	*M*	*h*_1_	*h*_2_	*f*′′(0)	−*θ*′(0)
0								−0.967323	0.997756
1								−1.010876	1.204641
2								−1.052693	1.384712
	1							−0.971748	1.019409
	2							−0.971748	0.914155
	3							−0.971748	0.838301
		0.5						−0.971748	1.002261
		1						−0.971748	0.953522
		1.5						−0.971748	0.897111
			0					−1.000742	1.292581
			0.5					−0.977007	1.085097
			1					−0.965996	0.933581
				0.1				−1.027406	0.984757
				0.2				−1.118266	0.913911
				0.3				−1.193398	0.837940
					1			−0.971748	1.019409
					2			−1.081534	0.959736
					3			−1.165272	0.907840
						0.5		−0.875050	0.988263
						1		−0.590892	0.871827
						1.5		−0.449540	0.792205
							0.5	−0.971748	0.925103
							1	−0.971748	0.632527
							1.5	−0.971748	0.480547

**Table 4:** Effects of non-dimensional quantities on *f*″(0),–*θ*′(0) for Methanol+CuO.

*λ*	*R*	*θ*_*w*_	*n*	*ϕ*_1_	*M*	*h*_1_	*h*_2_	*f*′′(0)	−*θ*′(0)
0								−0.948825	1.018470
1								−0.993286	1.227580
2								−1.036017	1.408668
	1							−0.953340	1.040397
	2							−0.953340	0.935769
	3							−0.953340	0.860242
		0.5						−0.953340	1.023306
		1						−0.953340	0.974511
		1.5						−0.953340	0.917535
			0					−0.971441	1.318493
			0.5					−0.956502	1.107259
			1					−0.949948	0.953006
				0.1				−0.953340	1.040397
				0.2				−0.866148	1.047195
				0.3				−0.772844	1.050301
					1			−0.953340	1.040397
					2			−1.055425	0.987021
					3			−1.134839	0.939898
						0.5		−0.859348	1.010119
						1		−0.582242	0.896578
						1.5		−0.443856	0.818370
							0.5	−0.953340	0.942355
							1	−0.953340	0.640545
							1.5	−0.953340	0.485161

**Figure 2:**
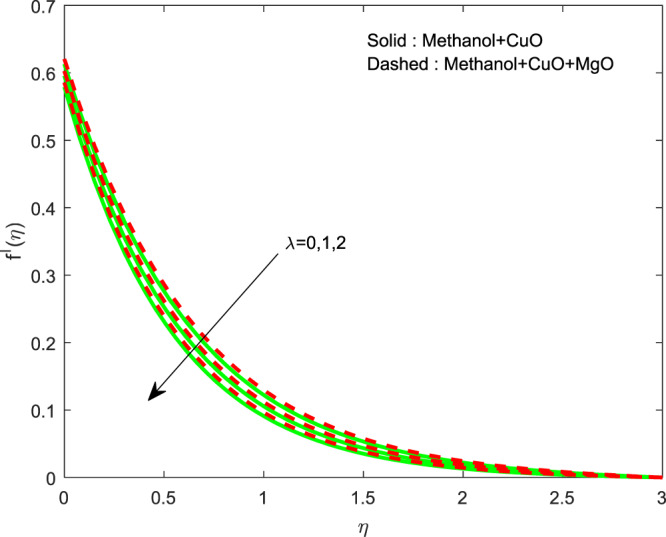
Variation of $\lambda$ on $f\text{'}(\eta )$.

**Figure 3:**
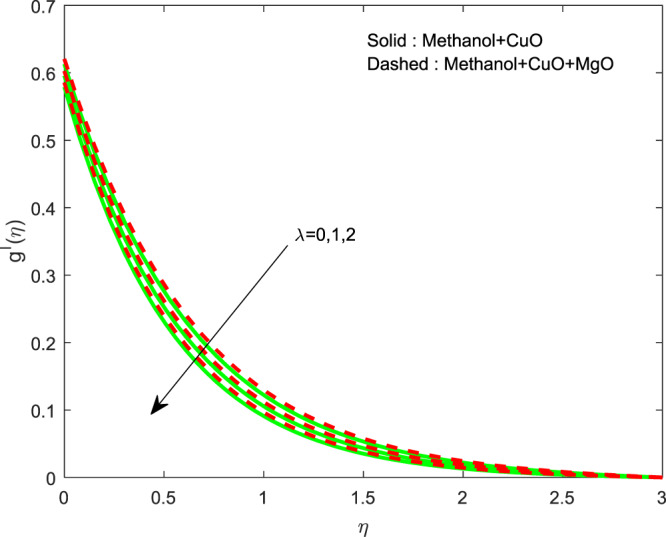
Variation of $\lambda$ on $g\text{'}(\eta )$.

**Figure 4:**
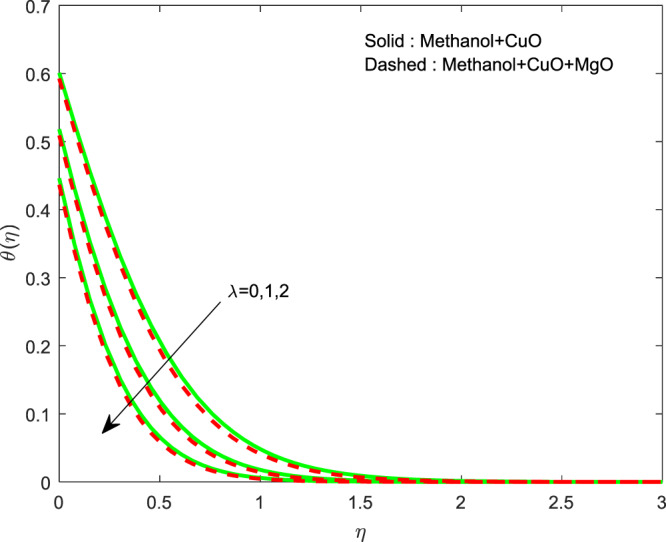
Variation of $\lambda$ on $\theta (\eta )$.

**Figure 5:**
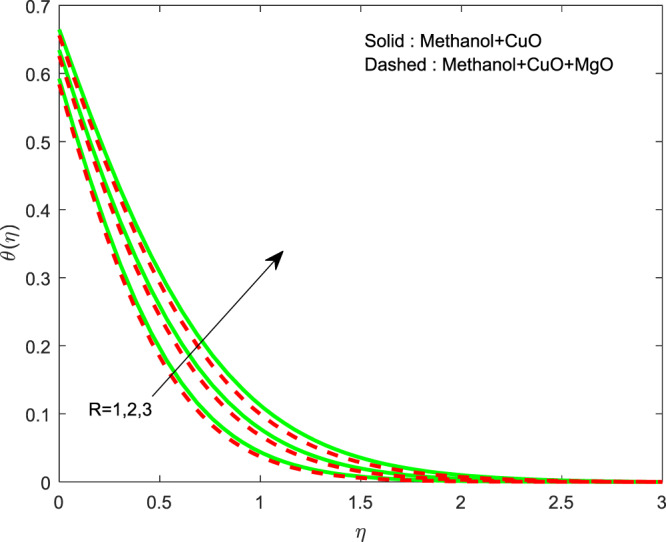
Variation of $R$ on $\theta (\eta )$.

The outcome of $\lambda$ on $f\text{'}(\eta ),g\text{'}(\eta )$ and $\theta (\eta )$ profiles are displayed in Figs. 2–4. The lessening of all the profiles has perceived by increase in $\lambda$. Generally, for escalations in wall thickness oppose the velocity. Temperature shrinks due to the transmission of heated particles is less in a denser area. The variation of $R$ on $\theta (\eta )$ is revealed in Fig. 5. It is clear that, thermal profile boosts by raising the thermal radiation. The growth in thermal profile is observed for escalations in $R$ which represents aid of an external source leads to rise in temperature.

**Figure 6:**
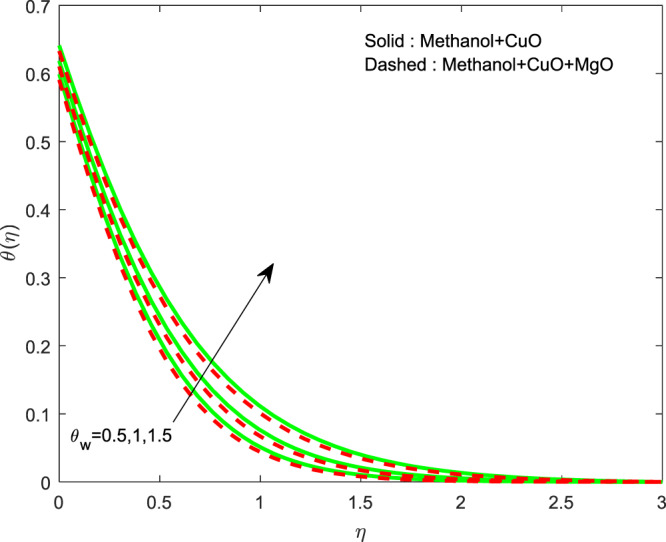
Variation of ${\theta }_{w}$ on $\theta (\eta )$.

**Figure 7:**
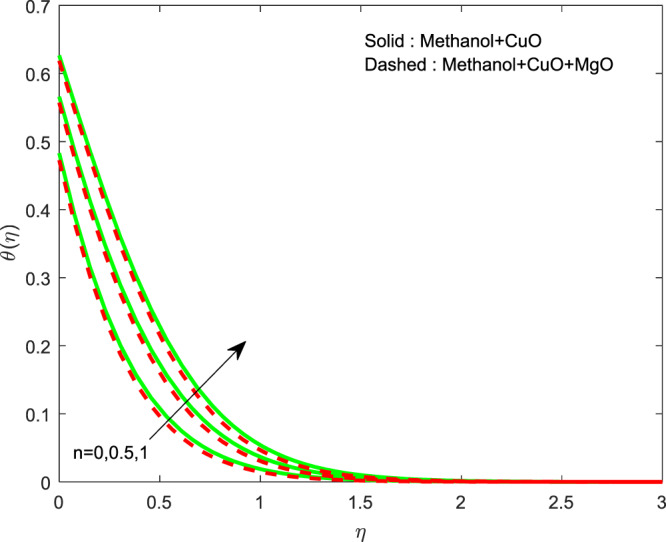
Variation of $n$ on $\theta (\eta )$.

**Figure 8:**
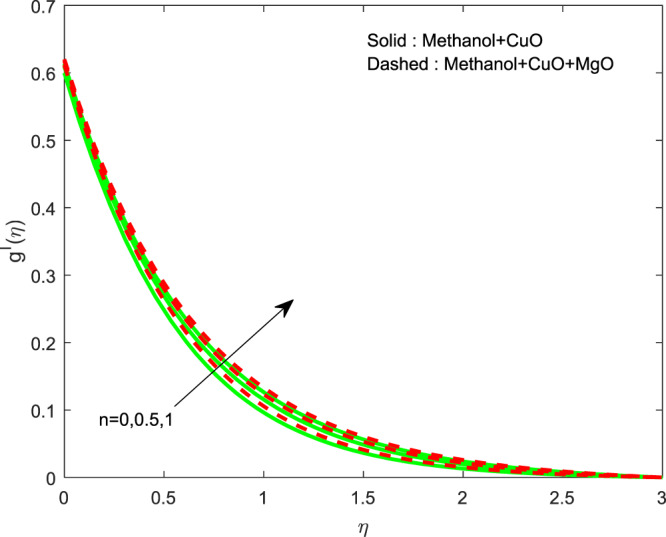
Variation of $n$ on $g\text{'}(\eta )$.

**Figure 9:**
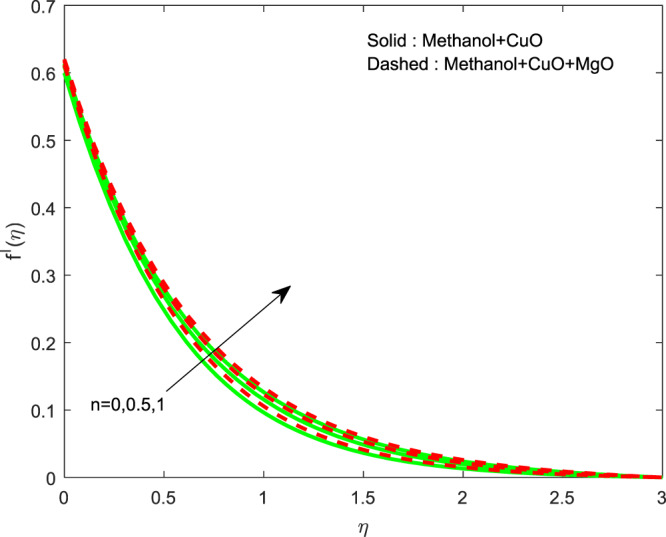
Variation of $n$ on $f\text{'}(\eta )$.

**Figure 10:**
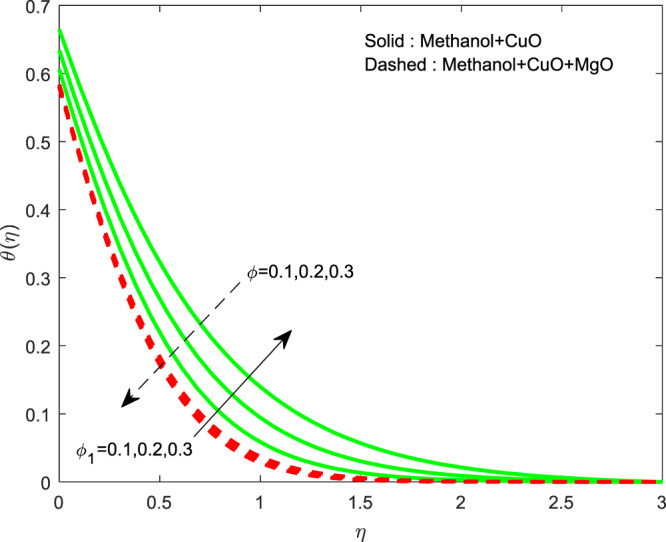
Variation of $\phi ,{\phi }_{1}$ on $\theta (\eta )$.

**Figure 11:**
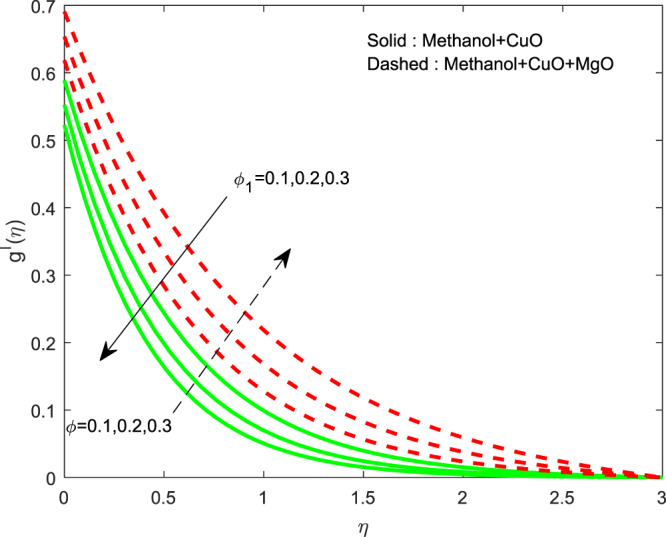
Variation of $\phi ,{\phi }_{1}$ on $g\text{'}(\eta )$.

**Figure 12:**
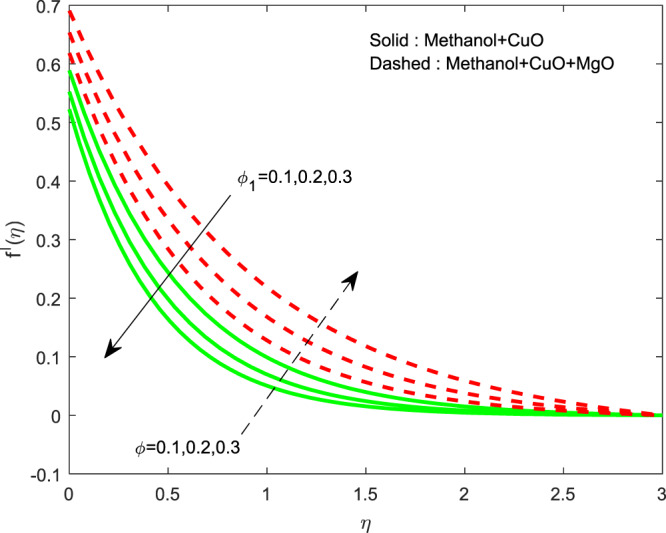
Variation of $\phi ,{\phi }_{1}$ on $f\text{'}(\eta )$.

The impact of ${\theta }_{w}$ on $\theta (\eta )$ is showed in Fig. 6. It illustrates that the thermal profile increases by evolving ${\theta }_{w}$. The variation of $n$ over $f\text{'}(\eta ),g\text{'}(\eta )$ and $\theta (\eta )$ profiles are discussed in Figs. 7–9, which reveals that all the profiles are increasing with rise in $n$. The object behind this is the thin boundary layer is executed by enhancing values of $n$. Figures 10–12 show the influence of $\phi ,{\phi }_{1}$ on $\theta (\eta ),f\text{'}(\eta )$ and $g\text{'}(\eta )$ profiles. It showed that the rise in $\phi$ increases the temperature profile, but the reverse action has perceived by the impacts of ${\phi }_{1}$. Also the rise in $\phi$ digresses the both velocity profiles but converse action have seen in existence of ${\phi }_{1}$. As volume fraction grows, then fluid turns to hike its density and get more concentration leads to reduce the velocity and hike temperature.

**Figure 13:**
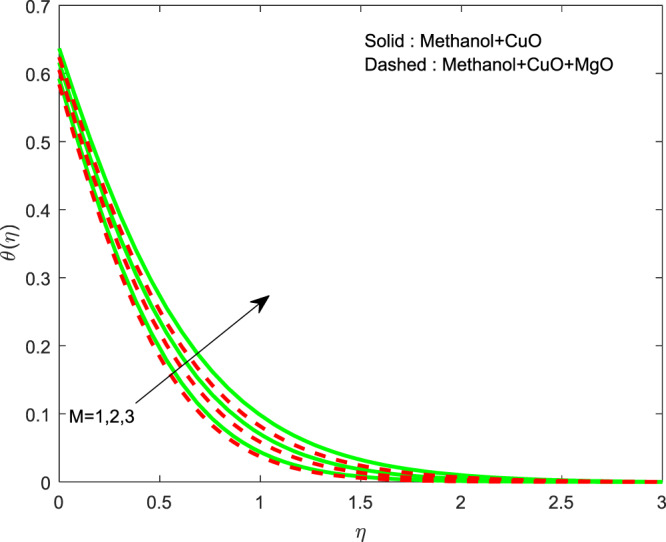
Variation of $M$ on $\theta (\eta )$.

**Figure 14:**
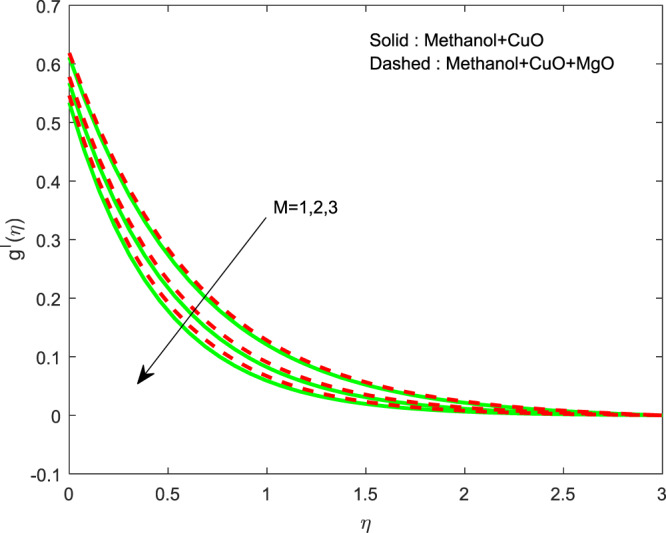
Variation of $M$ on $g\text{'}(\eta )$.

**Figure 15:**
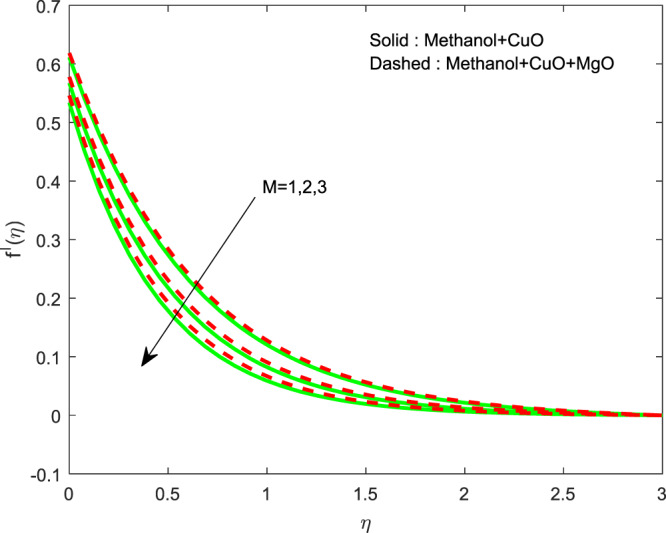
Variation of $M$ on $f\text{'}(\eta )$.

**Figure 16:**
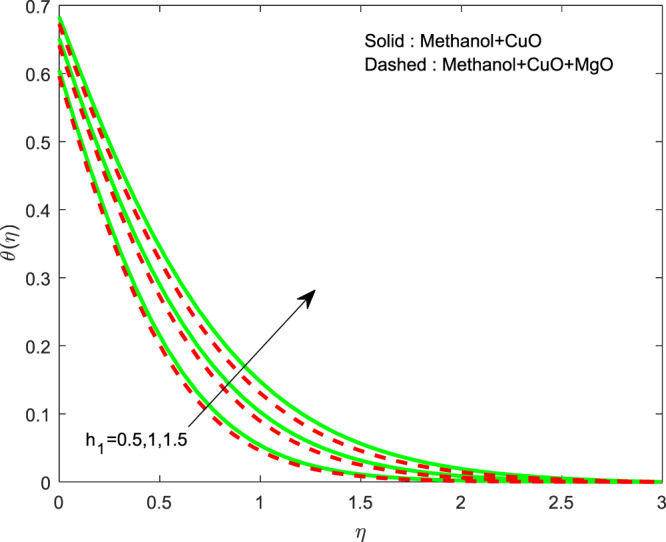
Variation of ${h}_{1}$ on $\theta (\eta )$.

**Figure 17:**
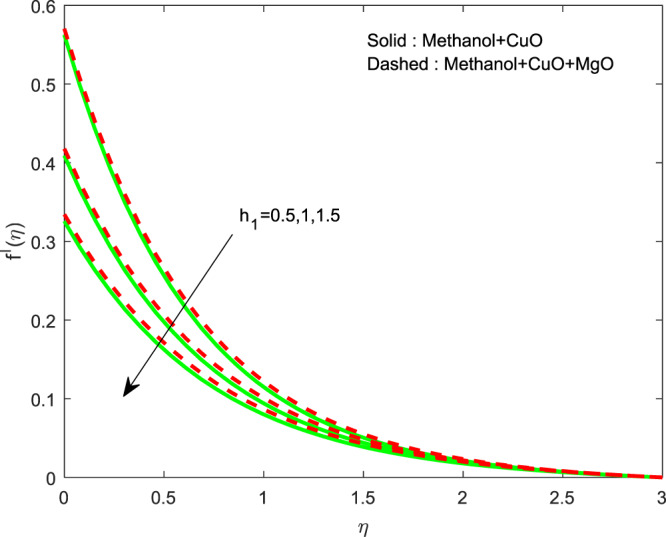
Variation of ${h}_{1}$ on $f\text{'}(\eta )$.

**Figure 18:**
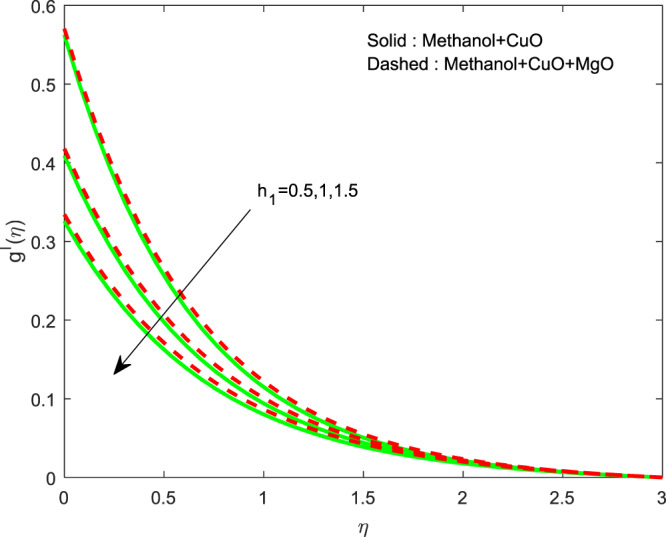
Variation of ${h}_{1}$ on $g\text{'}(\eta )$.

**Figure 19:**
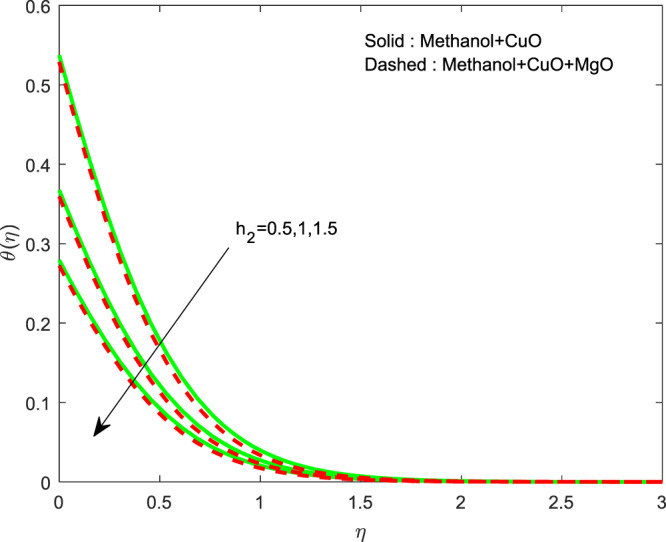
Variation of ${h}_{2}$ on $\theta (\eta )$.

Figures 13–15 represented the effect of $M$ on $\theta (\eta )$ and $f\text{'}(\eta ),g\text{'}(\eta )$ profiles. Obviously, increasing in temperature and a reduction in velocity has seen for developing $M$. The vitality of Lorentz force is gained for movements of electrically colloidal particles attracted by the effects of magnetic field. Therefore the velocity reduces and temperature enriches by Lorentz force. Figures (16–18) elucidates that an impact of ${h}_{1}$ on $\theta (\eta ),f\text{'}(\theta )$ and $g\text{'}(\theta )$ profiles. Raise in ${h}_{1}$ advances the thermal fields, but opposite action has seen for both the velocity. Cumulative the velocity slip parameter improves wall friction; this will cause the lessening in flow velocity. Generally, the slowdown in velocity turns to raise the temperature. Figure 19 displays the variant of ${h}_{2}$ over thermal field. It is noted that the temperature is falling due to hiking on ${h}_{2}$.

## Conclusions

Several scholars have pointed the assets of hybrid nanofluid are greater as associated with nanofluid. The persistence of this study is to examine the significance of heat transport with utilization of available nanoparticles to find greater hybrid nanofluid to enrich/decline the energy transfer. This study helps to identify the better combination of heat transfer material to enrich the thermal transport phenomenon. The conclusions are

- CuO+Methanol nanofluid shows better convection properties as related to CuO+MgO+Methanol hybrid nanofluid.
- The boundary thickness reduces by enhancing $n$.
- A Rise in ${h}_{1}$ advances the thermal field and decays the velocity.
- A Rise in ${h}_{2}$ intensifies the thermal field and monotonically deviate the Nusselt number.
- ${\theta }_{w}$ has propensity to lessen the heat rate.
- ${\phi }_{1}$ leads to magnify the flow and heat rate.
- CuO+MgO+Methanol combination may be used as an insulator.
- Application of the present study can be found in manufacturing industries.

## References

[1] Choi SUS, Eastman JA (1995). Enhancing thermal conductivity of fluids with nanoparticles. ASME International Mechanical Engineering Congress and Exposition.

[2] P.C.M. Kumar, K. Palanisamy, V. Vijayan, Stability analysis of heat transfer hybrid/water nanofluids, Materials Today: Proceedings. (2019). 10.1016/j.matpr.2019.06.743.

[3] Turcu R (2006). New polypyrrole-multiwall carbon nanotubes hybrid materials. Journal of Optoelectronics and Advanced Materials..

[4] Sarkar J, Ghosh P, Adil A (2015). A review on hybrid nanofluids: Recent research, development and applications. Renewable and Sustainable Energy Reviews..

[5] Shah TR, Ali HM (2019). Applications of hybrid nanofluids in solar energy, practical limitations and challenges: A critical review. Solar energy..

[6] Azwadi N (2016). Recent progress on hybrid nanofluids in heat transfer applications: A comprehensive review. International Communication in Heat and Mass Transfer..

[7] M. Hossein, M. Ghazvini, M. Sadeghzadeh, Utilization of hybrid nanofluids in solar energy applications: A review, Nano-Structures & Nano-Objects. 20 (2019). 10.1016/j.nanoso.2019.100386.

[8] Yarmand H (2016). Study of synthesis, stability and thermo-physical properties of graphene nanoplatelet / platinum hybrid nanofluid. International Communications in Heat and Mass Transfer..

[9] Huang D, Wu Z, Sunden B (2016). Effects of hybrid nanofluid mixture in plate heat exchangers. Experimental Thermal and Fluid Science..

[10] Labib MN, Nine J, Afrianto H, Chung H, Jeong H (2013). Numerical investigation on effect of base fluids and hybrid nanofluid in forced convective heat transfer. International Journal of Thermal Sciences..

[11] Waini I, Ishak A, Pop I (2019). Unsteady flow and heat transfer past a stretching/shrinking sheet in a hybrid nanofluid. International Journal of Heat and Mass Transfer..

[12] Ma Y, Mohebbi R, Rashidi MM, Yang Z (2019). MHD convective heat transfer of Ag-MgO/water hybrid nanofluid in a channel with active heaters and coolers. International Journal of Heat and Mass Transfer..

[13] Sheikholeslami M, Mehryan SAM, Shafee A, Sheremet MA (2019). Variable magnetic forces impact on magnetizable hybrid nanofluid heat transfer through a circular cavity. Journal of Molecular Liquids..

[14] Mehryan SAM, Kashkooli FM, Ghalambaz M, Chamkha AJ (2017). Free convection of hybrid Al 2 O 3 -Cu water nanofluid in a differentially heated porous cavity. Advanced Powder Technology..

[15] Acharya N, Bag R, Kundu PK (2019). Influence of Hall current on radiative nanofluid flow over a spinning disk: A hybrid approach. Physica E: Low-dimensional Systems and Nanostructures..

[16] Reza H, Shahriari A (2018). MHD natural convection of hybrid nanofluid in an open wavy cavity. Results in Physics..

[17] Fang T, Zhang J, Zhong Y (2012). Boundary layer flow over a stretching sheet with variable thickness. Applied Mathematics and Computation..

[18] Khader MM, Megahed AM (2013). Numerical solution for boundary layer flow due to a nonlinearly stretching sheet with variable thickness and slip velocity. The European Physical Journal Plus..

[19] Khan M, Malik MY, Salahuddin T (2017). Heat generation and solar radiation effects on Carreau nanofluid over a stretching sheet with variable thickness: Using coefficients improved by Cash and Carp. Results in Physics..

[20] Sandeep N (2016). MHD radiative flow and heat transfer of a dusty nanofluid over an exponentially stretching surface. Engineering Science and Technology, an International Journal..

[21] Hayat T, Hussain Z, Muhammad T, Alsaedi A (2016). Effects of homogeneous and heterogeneous reactions in flow of nanofluids over a nonlinear stretching surface with variable surface thickness. Journal of Molecular Liquids..

[22] Babu MJ, Sandeep N (2016). 3D MHD slip flow of a nanofluid over a slendering stretching sheet with thermophoresis and Brownian motion effects. Journal of Molecular Liquids..

[23] Babu MJ, Sandeep N, Ali ME, Nuhait AO (2017). Magnetohydrodynamic dissipative flow across the slendering stretching sheet with temperature dependent variable viscosity. Results in Physics..

[24] C. Sulochana, S.P. Samrat, N. Sandeep Boundary layer analysis of an incessant moving needle in MHD radiative nanofluid with joule heating, International Journal of Mechanical Sciences. 128–129 (2017) 326–331. 10.1016/j.ijmecsci.2017.05.006.

[25] Sulochana C, Samrat SP, Sandeep N (2018). Numerical investigation of magnetohydrodynamic (MHD) radiative flow over a rotating cone in the presence of Soret and chemical reaction, Propulsion and Power. Research..

[26] Usman M, Hamid M, Zubair T, Haq RU, Wang W (2018). Cu-Al 2 O 3 / Water hybrid nanofluid through a permeable surface in the presence of nonlinear radiation and variable thermal conductivity via LSM. International Journal of Heat and Mass Transfer..

[27] Nayak MK, Sher N, Pandey VS, Hayat Z, Tripathi D (2017). 3D free convective MHD flow of nanofluid over permeable linear stretching sheet with thermal radiation. Powder Technology..

[28] Khan MI, Hafeez MU, Hayat T, Khan MI, Alsaedi A (2020). Computer Methods and Programs in Biomedicine Magneto rotating flow of hybrid nanofluid with entropy generation. Computer Methods and Programs in Biomedicine..

[29] Reddy JVR, Sugunamma V, Sandeep N, Sulochana C (2016). Influence of chemical reaction, radiation and rotation on MHD nanofluid flow past a permeable flat plate in porous medium. Journal of the Nigerian Mathematical Society..

[30] S.P. Samrat, C. Sulochana, G.P. Ashwinkumar, Impact of Thermal Radiation on an Unsteady Casson Nanofluid Flow Over a Stretching Surface, International Journal of Applied and Computational Mathematics. **123**10.1007/s40819-019-0606-2 (2019).

[31] Iqbal Z, Akbar NS, Azhar E, Maraj EN (2018). Performance of hybrid nanofluid (Cu-CuO / water) on MHD rotating transport in oscillating vertical channel inspired by Hall current and thermal radiation. Alexandria Engineering Journal..

[32] Khader M, Megahed AM (2013). Numerical solution for boundary layer flow due to a nonlinearly stretching sheet with variable thickness and slip velocity. The European Physical Journal Plus.

[33] Vinod Kumar G, Verma SVK, Kumar RVMSSK (2019). Unsteady three-dimensional MHD nanofluid flow over a Stretching sheet with variable wall thickness and slip effects, Int. J. of Applied Mechanics and Engineering.

[34] Anjali Devi, S.P. & Prakash, M., Thermal radiation effects on hydromagnetic flow over a slendering stretching sheet, J. Braz. Soc. Mech. Sci. Eng., 10.1007/s40430-015-0315-7 (2015).

[35] Sankar M, Venkatachalappa M, Shivakumar IS (2006). Effect of magnetic field on natural convection in a vertical cylindrical annulus. International Journal of Engineering Science.

[36] Ramesh, Y D, G. K., Roopa, G. S. & Sankar, M. Navier’s slip condition on time dependent Darcy-Forchheimer nanofluid using spectral relaxation method,Journal of Central South University **26** (7), 2000–2010.

[37] Sankar, M., Girish, N. & Siri, Z. Fully developed magnetoconvective heat transfer in vertical double-passage porous annuli, Flow and transport in subsurface environment, 217–249 (2018).

[38] Jagadeesha RD, Prasanna BMR, Younghae D, Sankar M (2017). Natural convection in an inclined parallelogrammic porous enclosure under the effect of magnetic field,Journal of Physics. Conference Series.

[39] Sankar M, Venkatachalappa M, Do Younghae (2011). Effect of magnetic field on the buoyancy and thermocapillary driven convection of an electrically conducting fluid in an annular enclosure. International Journal of Heat and Fluid Flow.

[40] Venkatachalappa M, Younghae D, Sankar M (2011). Effect of magnetic field on the heat and mass transfer in a vertical annulus. International Journal of Engineering Science.

